# Screening and Toxigenic Corynebacteria Spread

**DOI:** 10.3201/eid1203.050601

**Published:** 2006-03

**Authors:** Natasha S. Crowcroft, Joanne M. White, Androulla Efstratiou, Robert George

**Affiliations:** * Health Protection Agency Centre for Infections, London, United Kingdom

**Keywords:** diphtheria, secondary cases, surveillance, corynebacteria

**To the Editor:** Diphtheria is rare in countries with high vaccination coverage, but as seen in Europe in recent decades, control can disintegrate rapidly. When diphtheria is rare, surveillance is challenging because clinicians have no experience with the infection, and disease may be mild or atypical in vaccinated persons (*1*). Clinicians may give inadequate information to laboratories, and appropriate investigations may not be performed. Identifying cases is facilitated if all throat swabs from patients with pharyngitis are screened by laboratories for corynebacteria, but this procedure is expensive and time-consuming. To help balance priorities in diphtheria surveillance, we evaluated the potential benefits of microbiologic screening in preventing secondary spread of toxigenic corynebacteria in England and Wales and estimated the possible consequences of not detecting a case.

The mean number of secondary cases that might occur per index case if screening is not undertaken depends on the mean number of contacts and attack rates, vaccine coverage and efficacy, and duration of protection. Some of these factors are not known precisely, so we estimated them within plausible ranges of values. We varied the number of contacts per case-patient from 2 to 20. Secondary attack rates in susceptible persons are difficult to estimate and distinguish from carriage rates (*2*), and we varied these from 5% to 50%. Vaccine efficacy in children was varied from 50% to 95%. We estimated the susceptibility of UK adults at 40% (*3*), vaccination coverage in children at 95% (*4*), and case-fatality ratio at 6% to 10% (*5*). For simplicity, the ratio of adults to children among contacts was assumed to be 1:1. We assumed that without specific microbiologic identification of cases, no intervention would take place and that intervention to protect contacts is 100% effective. Such intervention includes early treatment and isolation of cases, chemoprophylaxis, and booster vaccination of contacts. The number of cases that need to be detected to prevent 1 secondary case for different numbers of contacts and attack rates was calculated as the inverse of the number of secondary cases that would result from each case not detected by screening.

The number of cases that must be detected by microbiologic screening to prevent 1 secondary case was most affected by varying the number of contacts per patient and the secondary attack rate (Figure). If one assumes vaccine efficacy of 95%, an attack rate in susceptible contacts of 5%, and 4 contacts per patient, 1 secondary case is prevented for every 18 cases detected; if attack rates are 30%, then 1 secondary case is prevented for <5 index cases detected. If vaccine efficacy was 50%, the number of cases that would need to be detected to prevent 1 secondary case would fall from 18 to <10 cases for a mean of 4 contacts per case and secondary attack rates of 5%.

**Figure:**
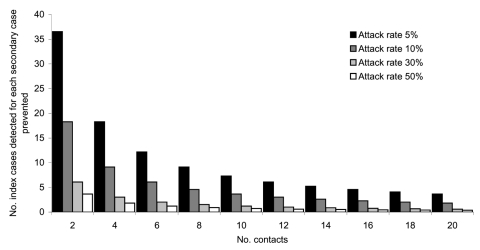
Number of cases needed to detect to prevent 1 secondary case.

For the 53 toxigenic strains of corynebacteria detected in England and Wales from 1993 to 2000, an estimated 2–10 secondary cases would have been prevented if attack rates were 5% and each patient had 4 contacts. The number of index cases needed to be detected to prevent 1 death (assuming 6%–10% case-fatality ratio) would have been 150–180 with attack rates of 5% and 50–83 with attack rates of 30%. Thus, deaths were not likely to have been prevented during this period by screening.

Are the parameter estimates valid? We focused on secondary cases, but spread in outbreaks may be exponential, so the effect of missing cases may be greater once tertiary cases and further spread are taken into account. Vaccination coverage may be higher or lower in different risk groups. Secondary attack rates in the literature are reported from outbreaks and regions with vulnerable populations during periods of high incidence and may not apply in affluent countries with high coverage and may be <5%. Adult protection may be better than indicated by serosurveys and may have improved in the United Kingdom with use since 1994 of combined tetanus-diphtheria toxoid vaccine instead of tetanus toxoid for injuries (*5*).

Outbreaks are not reported from countries without routine screening (*1*), which indicates that some of our assumptions and estimates may be incorrect. Alternatively, this fact may indicate defective surveillance; countries that do not detect primary cases may not detect secondary cases.

Surveillance for diphtheria in European Union member states varies widely (*1*). Only 5 of 19 reporting countries screen throat swabs routinely for corynebacteria, raising doubts about the quality of surveillance. The absence of reports of diphtheria may not reflect the absence of disease or of circulating toxigenic corynebacteria. Our results show the possible consequences of not detecting such infections and help demonstrate the public health priority of diphtheria surveillance.

## References

[1] De Zoysa A, Efstratiou A. Eighth international meeting of the European Laboratory Working Group on Diphtheria and the Diphtheria Surveillance Network—June 2004: progress is needed to sustain control of diphtheria in European Region. Euro Surveill [serial on the Internet]. 2004 Nov [cited 2006 Jan 25]. Available from http://www.eurosurveillance.org/em/v09n11/0911-227.asp 10.2807/esm.09.11.00489-en 29183467

[2] Vitek CR, Wharton M. Diphtheria in the former Soviet Union: reemergence of a pandemic disease. Emerg Infect Dis. 1998;4:539–50. 10.3201/eid0404.980404 9866730 PMC2640235

[3] Edmunds WJ, Pebody RG, Aggerback H, Baron S, Berbers G, Conyn-van Spaendonck MA, The sero-epidemiology of diphtheria in western Europe. European Sero-Epidemiology Network project. Epidemiol Infect. 2000;125:113–25. 10.1017/S0950268899004161 11057967 PMC2869577

[4] Department of Health. Statistical bulletin. NHS immunisation statistics, England: 2003–04. London. 2004. Available at http://www.dh.gov.uk/assetRoot/04/09/95/77/04099577.pdf

[5] Galazka A. The changing epidemiology of diphtheria in the vaccine era. J Infect Dis. 2000;181:S2–9. 10.1086/315533 10657184

